# Optimization of Surface-Enhanced Raman Spectroscopy Detection Conditions for Interaction between Gonyautoxin and Its Aptamer

**DOI:** 10.3390/toxins14010049

**Published:** 2022-01-11

**Authors:** Yan Liu, Lijuan He, Yunli Zhao, Yongbing Cao, Zhiguo Yu, Feng Lu

**Affiliations:** 1 Department of Pharmaceutical Analysis, College of Pharmacy, Naval Medical University, Shanghai 200433, China; mcliuyan@hotmail.com; 2 Shanghai Key Laboratory for Pharmaceutical Metabolite Research, Naval Medical University, Shanghai 200433, China; 3 Department of Pharmaceutical Analysis, School of Pharmacy, Shenyang Pharmaceutical University, Shenyang 110016, China; hlj5826@163.com (L.H.); yunli76@163.com (Y.Z.); 4 Institute of Vascular Disease, Shanghai TCM-Integrated Hospital, Shanghai University of Traditional Chinese Medicine, Shanghai 200082, China

**Keywords:** aptamer, SERS, buffer solution, interaction, gonyautoxin, silver colloids

## Abstract

This study aimed to optimize the detection conditions for surface-enhanced Raman spectroscopy (SERS) of single-stranded DNA (ssDNA) in four different buffers and explore the interaction between gonyautoxin (GTX1/4) and its aptamer, GO18. The influence of the silver colloid solution and MgSO_4_ concentration (0.01 M) added under four different buffered conditions on DNA SERS detection was studied to determine the optimum detection conditions. We explored the interaction between GTX1/4 and GO18 under the same conditions as those in the systematic evolution of ligands by exponential enrichment technique, using Tris-HCl as the buffer. The characteristic peaks of GO18 and its G-quadruplex were detected in four different buffer solutions. The change in peak intensity at 1656 cm^−1^ confirmed that the binding site between GTX1/4 and GO18 was in the G-quadruplex plane. The relative intensity of the peak at 1656 cm^−1^ was selected for the GTX1/4–GO18 complex (I_1656_/I_1099_) to plot the ratio of GTX1/4 in the Tris-HCl buffer condition (including 30 μL of silver colloid solution and 2 μL of MgSO_4_), and a linear relationship was obtained as follows: Y = 0.1867X + 1.2205 (R^2^ = 0.9239). This study provides a basis for subsequent application of SERS in the detection of ssDNA, as well as the binding of small toxins and aptamers.

## 1. Introduction

Aptamers are single-stranded DNAs (ssDNAs) capable of binding to the corresponding ligands with high affinity and strong specificity. They have been isolated via the systematic evolution of ligands by exponential enrichment (SELEX) [1]. SELEX technology allowed for the screening of a large number of aptamers that can act on proteins [2], toxins [3,4], ions [5], drug molecules [6], and even cells [7], which are widely used in various fields, such as analysis, detection, sensing, clinical treatment, and diagnosis. Aptamers can combine with ligands through hydrogen bonds, van der Waals forces, and other forces to form secondary structures, such as hairpin, helix, and G-quadruplex [8,9], with excellent selectivity and reproducibility. Compared with antibodies, aptamers have the characteristics of good thermal stability, strong salt tolerance, low cost, and easy synthesis [10,11], which makes aptamers more advantageous for analysis and detection.

In recent years, the main methods used to study the interaction between aptamers and small molecules has included isothermal titration calorimetry [12], surface plasmon resonance (SPR) [13], microscale thermophoresis (MST) [14], biolayer interferometry (BLI) [15], nuclear magnetic resonance (NMR) [16], circular dichroism (CD) [17], and molecular dynamics (MD) [18]. These methods provide reliable kinetic or thermodynamic information about the interaction between aptamers and small-molecule ligands. However, they have certain limitations. For example, SPR, MST, and BLI require labeling and immobilization of ligands or aptamers; CD spectroscopy hardly provides the base sequence or structural information of the DNA chain. NMR can obtain accurate structural information, but it requires many samples. GTX1/4, studied in this paper, is one of the most potent paralytic toxins in marine organisms, with a molecular weight of 411.35 [19]. GO18, the aptamer of GTX1/4, was obtained by the Wang group through SELEX [20], and GO18 was used to construct a BLI biosensor to detect the dissociation constant of GTX1/4 and GO18, and the measured Kd value was 17.7 nM. Song et al. [21] used MD to calculate the interaction mode between GO18 and GTX1/4. However, they only used MST to detect the affinity of the GO18 and GTX1/4, and there is no experimental method to directly verify the mechanism of their interaction. Herein, we aimed to find a more general method, using small amounts of samples, that helps to detect the interaction between the aptamer and toxin in various buffer solutions.

Surface-enhanced Raman spectroscopy (SERS) is an analytical method based on Raman spectroscopy. Its essence is the scattering caused by inelastic collisions between photons and material molecules caused by monochromatic light irradiating the surface molecules of the material [22,23,24,25]. SERS is a powerful biochemical fingerprint-recognition technology that can obtain a large number of amplified Raman signals from the Raman-active analyte molecules attached to some rough, precious metal surfaces. It requires a small amount of sample and greatly improves the detection sensitivity. It is widely used in fields such as food safety, environmental monitoring, and drug monitoring [26,27,28]. SERS has become a powerful analytical tool for rapid detection and structural characterization of DNA, owing to its ultra-high sensitivity and ability to provide inherent chemical fingerprint information. SERS-based DNA detection can be classified into two categories. The first is an indirect method that makes use of labeling molecules to modify and detect the target DNA [29,30]. The second method directly obtains the inherent SERS spectra of DNA without labeling [31,32]. In recent years, direct detection of DNA without labels has been rapidly developed, and it is currently the most commonly used detection method. For example, in 2015, Xu et al. [33] developed iodine-modified silver nanoparticles and introduced MgSO_4_ into silver nanoparticles to promote the aggregation of silver nanoparticles, thus obtaining SERS signals of highly repeatable ssDNA and achieving highly sensitive detection of single-base mismatch. Li et al. [34] used silver nanoparticles to study the secondary structure of DNA and successfully detected the structural characteristics of the DNA i-motif. This method significantly improved the application scope of SERS, which is not only limited to ssDNA and single-base detection but also applicable to the detection of polymeric DNA. In recent years, in studies reporting SERS detection of ssDNA, the main solvent was water. Xu et al. [33], for example, established a reliable and important method of label-free DNA detection based on silver nanoparticles in aqueous solutions close to physiological conditions. In terms of interaction, phosphate and Tris-HCl are the main buffer solutions in the detection system [35]. In this case, it is very important and meaningful to establish a detection system that can detect the interaction between the aptamer and the toxin and be performed in a variety of buffer solutions.

**Figure 1:**
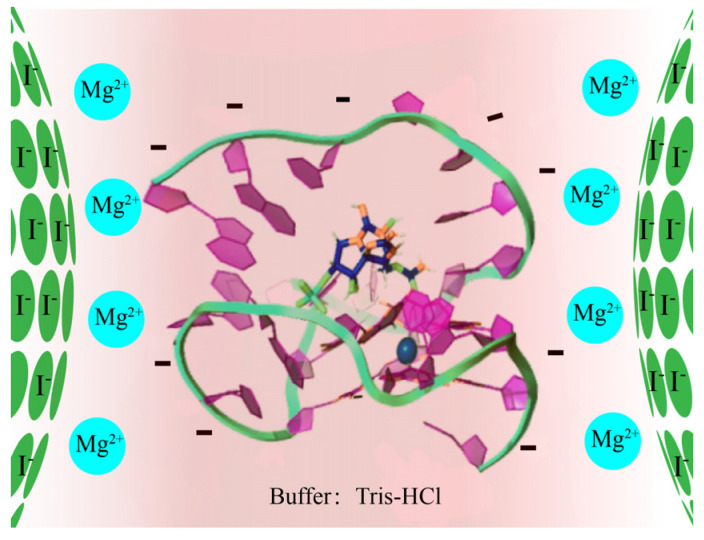
Schematic diagram of the SERS enhancement mechanism for the GO18–GTX1/4 complex.

In this study, the conditions for the rapid, sensitive, and label-free SERS detection of GTX1/4 aptamers under four different buffered conditions (water, acetate, phosphate, and Tris-HCl) were established by optimizing the amount of silver colloid solution and MgSO_4_ using silver-gel nanoparticles as the enhanced base and MgSO_4_ as the coagulant. This study provides a reference method for SERS detection of aptamers. The interaction between GTX1/4 and its aptamer was investigated using Tris-HCl buffer conditions consistent with SELEX. The SERS enhancement mechanism of the GO18–GTX1/4 complex is shown in Figure 1.

## 2. Results and Discussion

### 2.1. SERS Optimization in Four Kinds of Buffers

SERS detection of ssDNA [33,36] was performed as follows. The silver colloid solution was concentrated via centrifugation to increase the number of “hot spots.” Considering that excess citrate was present on the surface of the silver colloid solution after concentrating the solution, Kalium Iodidum (KI) was added as the “cleaning agent” to clean the surface of the silver colloid solution and, a layer of I^-^ was used to negatively charge the surface of the silver colloids. As the phosphoric-acid skeleton of DNA was negatively charged, it could not get close to the negatively charged silver colloid particle “hot spot” and did not generate a good SERS signal. Therefore, MgSO_4_ was added as a coagulant, acting as a bridge, bringing negatively charged silver colloid particles and negatively charged DNA molecules close to each other so that DNA molecules were close to the silver colloid particle “hot spot.” When MgSO_4_ solution was added in excess, the color of the sample solution became dark. With continuous mixing, the solution exhibited an aggregation phenomenon, and a stable SERS signal for the DNA could not be obtained. When a small amount of MgSO_4_ solution was used, it was not enough to bind as much DNA as possible to the surface of the silver colloids and generate high-intensity signals. Therefore, during the actual operation, the DNA solution was added to the silver colloid solution to allow the DNA molecules to surround the silver glue particles, and only then, the MgSO_4_ solution was added. On the one hand, in the presence of Mg^2+^, silver colloids form agglomerations, forming more “hot spots”. On the other hand, they bring DNA molecules to the surface of the silver colloids, thereby generating SERS signals. The amount of MgSO_4_ added plays an important role in SERS detection.

#### 2.1.1. Water as the Solvent

**Figure 2:**
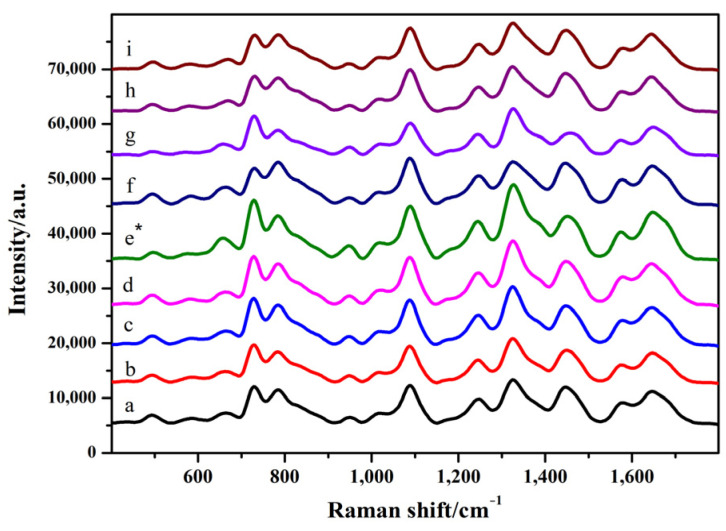
Surface-enhanced Raman spectroscopy (SERS) spectra of GO18 in water. a–c: 4 μL of silver colloid solution; 1, 2, and 3 μL of MgSO_4_, respectively. d–f: 5 μL of silver colloid solution; 1, 2, and 3 μL of MgSO_4_, respectively. g–i: 6 μL of silver colloid solution; 1, 2, and 3 μL of MgSO_4_, respectively. “*” means the optimal condition.

As shown in Figure 2 and Table S1 (see Supplementary Materials), when 4 μL of the silver colloid solution was added, the SERS intensity increased with an increase in the amount of MgSO_4_. When 5 μL of silver colloid solution and 2 μL of MgSO_4_ were added, the peak intensities at 1099 and 1656 cm^−1^ increased. When the amount of silver colloid solution was 6 μL, the intensity of the SERS pattern did not change significantly and was weakened with the increase in the amount of MgSO_4_. Considering the intensity and characteristic peaks of SERS chromatograms, the optimum conditions for SERS detection were as follows: 5 μL of silver colloid solution and 2 μL of MgSO_4_.

#### 2.1.2. Sodium Acetate Buffer as the Solvent

**Figure 3:**
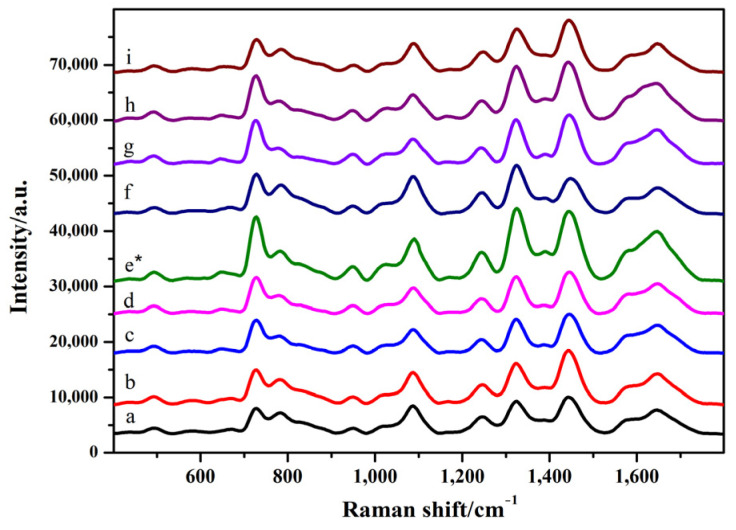
SERS spectra of GO18 in sodium acetate solution. a–c: 4 μL of silver colloid solution; 1, 2, and 3 μL of MgSO_4_, respectively. d–f: 5 μL of silver colloid solution; 1, 2, and 3 μL of MgSO_4_, respectively. g–i: 6 μL of silver colloid solution; 1, 2, and 3 μL of MgSO_4_, respectively. “*” means the optimal condition.

As shown in Figure 3 and Table S2 (see Supplementary Materials), when 4 μL of the silver colloid solution was added, the intensity of the characteristic peak did not change significantly with the increase in the amount of MgSO_4_. When 5 μL of silver colloid solution and 2 μL of MgSO_4_ were added, the peak intensities at 1099, 1326, and 1656 cm^−1^ increased. When the amount of silver colloid solution was 6 μL, the intensity of the SERS pattern did not change significantly and was weakened with the increase in the amount of MgSO_4_. Considering the intensity and characteristic peaks of SERS chromatograms, the optimum conditions for SERS detection were as follows: 5 μL of silver colloid solution and 2 μL of MgSO_4_.

#### 2.1.3. Sodium Phosphate Buffer as the Solvent

**Figure 4:**
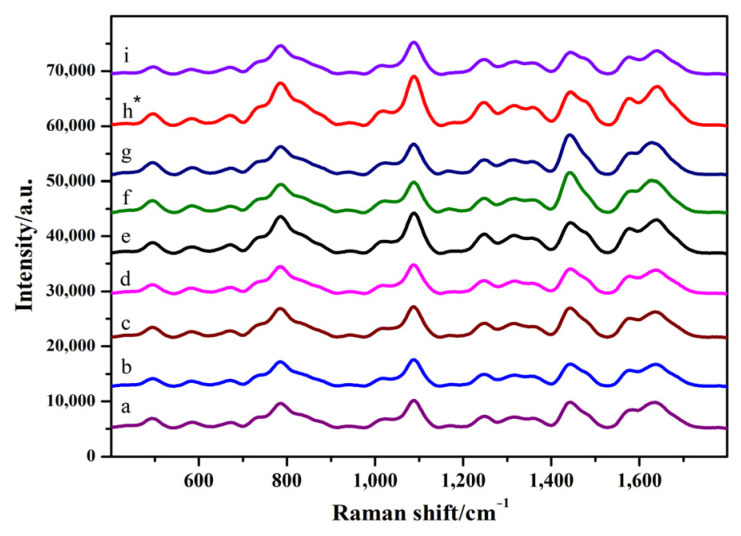
SERS spectra of GO18 in sodium phosphate solution. a–c: 4 μL of silver colloid solution; 1, 2, and 3 μL of MgSO_4_, respectively. d–f: 5 μL of silver colloid solution; 1, 2, and 3 μL of MgSO_4_, respectively. g–i: 6 μL of silver colloid solution; 1, 2, and 3 μL of MgSO_4_, respectively. “*” means the optimal condition.

As shown in Figure 4 and Table S3 (see the Supplementary Materials), when the amount of the silver colloid solution was 5 μL, the SERS intensity increased with an increase in the amount of MgSO_4_. When the amount of silver colloid solution was 6 μL and that of MgSO_4_ was 2 μL, the strength of the SERS peak generated by the stretching vibration of the PO^2-^ skeleton at 789 and 1099 cm^−1^ was significantly increased, and the strength of the peaks at 1581 and 1656 cm^−1^ related to the hydrogen bond formed in the G-quadruplex was significantly increased. Considering the intensity and characteristic peaks of the SERS chromatograms, the optimum conditions for SERS detection were as follows: 6 μL of silver colloid solution and 2 μL of MgSO_4_.

#### 2.1.4. Tris-HCl Buffer as the Solvent

**Figure 5:**
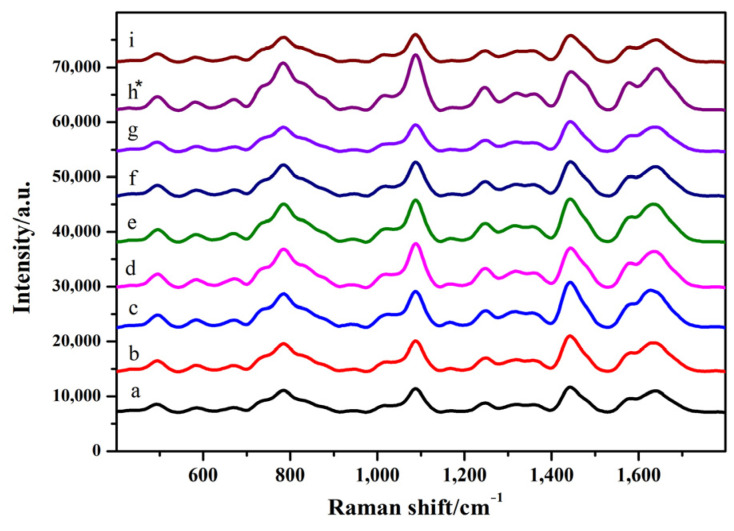
SERS spectra of GO18 in Tris-HCl. a–c: 5 μL of silver colloid solution; 1, 2, and 3 μL of MgSO_4_, respectively. d–f: 6 μL of silver colloid solution; 1, 2, and 3 μL of MgSO_4_, respectively. g–i: 7 μL of silver colloid solution; 1, 2, and 3 μL of MgSO_4_, respectively. “*” means the optimal condition.

As shown in Figure 5 and Table S4 (see Supplementary Materials), when the amount of the silver colloid solution was 5 μL, the SERS intensity did not change significantly and was weak with the increase in the amount of MgSO_4_. When the amount of silver colloid solution was 6 μL, the strength of the SERS pattern weakened gradually with an increase in the amount of MgSO_4_. When the amount of silver colloid solution was 7 μL and that of MgSO_4_ was 2 μL, the peak intensities at 1099 and 1656 cm^−1^ increased. Therefore, the optimized SERS detection conditions were as follows: 7 μL of silver colloid solution and 2 μL of MgSO_4_.

It can be observed from the figures that under water- and acetate-buffered conditions, the amounts of silver colloid solution and MgSO_4_ were the same, and the SERS spectra of GO18 were very similar. Furthermore, under phosphate- and Tris-HCl-buffered conditions, the SERS spectra of GO18 were very similar. This might be because water- and acetate-buffered conditions were relatively simple, whereas phosphate buffer contained phosphate ions and Tris-HCl was used as a polyhydric alcohol compound, which might affect the aptamer being brought to the “hot spots” generated by the silver colloids. The SERS characteristic peak of the aptamer changed in intensity and peak shape.

According to the results, based on the SERS detection conditions of silver colloids as an enhanced substrate and MgSO_4_ as the coagulant, GO18 and its G-quadruplex characteristic peaks could be detected under four different buffered conditions. Moreover, even if the buffer conditions changed, their effect on SERS detection of aptamers was small, indicating that the detection conditions were relatively stable, providing a reference for SERS detection of aptamers under other buffered conditions and laying a foundation for the subsequent study of the interaction mechanism between GTX1/4 and GO18.

### 2.2. The Interaction Mechanism between GTX1/4 and GO18

#### 2.2.1. Establishment and Optimization of SERS Detection Conditions

As mentioned above, SERS technology established under four buffered conditions can help obtain the relevant characteristic information of GO18 and its G-quadruplex. In the interaction study, considering that SELEX screening of the GTX1/4 aptamer was performed in the Tris-HCl buffer system, to truly reflect the interaction between GO18 and GTX1/4 in the subsequent SERS analysis, SERS detection was performed in the same Tris-HCl buffer system as was used in SELEX screening.

As the actual SELEX Tris-HCl buffer system (20 mM Tris-HCl, 100 mM NaCl, 5 mM KCl, 2 mM MgCl_2_, pH 7.5) contained Na^+^, K^+^, and Mg^2+^ cations, in addition to Tris-HCl, the presence of these ions affected SERS detection. Therefore, we further optimized SERS conditions and the subsequent analysis of the interaction between GO18 and GTX1/4 to meet the requirements of GO18 detection using the Tris-HCl buffer system.

#### 2.2.2. SERS Condition Optimization

We optimized the SERS conditions for GO18 at certain concentrations and the amount of samples, mainly optimizing the amounts of the silver colloid solution (20, 30, and 40 μL) and MgSO_4_ solution (1, 2, and 3 μL).

**Figure 6:**
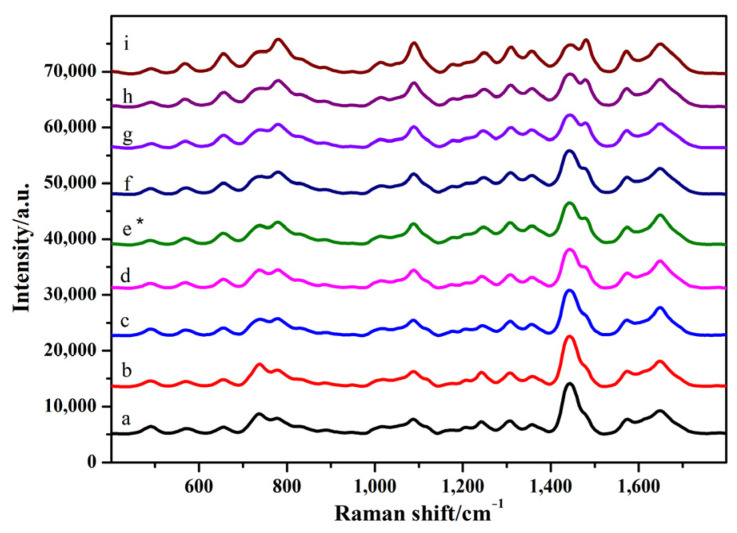
SERS spectra of GO18 in Tris-HCl. a–c: 20 μL of silver colloid solution; 1, 2, and 3 μL of MgSO_4_, respectively. d–f: 30 μL of silver colloid solution; 1, 2, and 3 μL of MgSO_4_, respectively. g–i: 40 μL of silver colloid solution; 1, 2, and 3 μL of MgSO_4_, respectively. “*” means the optimal condition.

As shown in Figure 6 and Table S5 (see Supplementary Materials), when the amount of silver colloid solution was 20 μL and that of MgSO_4_ was 1, 2, and 3 μL, the SERS spectra were relatively similar. When the amount of silver colloid solution was 30 μL and that of MgSO_4_ was 2 μL, the peak intensity at 1656 cm^−1^ increased. When the amount of silver colloid solution was 40 μL and that of MgSO_4_ was 3 μL, the peak intensities at 1099 and 1656 cm^−1^ increased, but their ratio did not.

As a subsequent study was performed to detect DNA, especially the structural characteristic information of the G-quadruplex, the SERS detection conditions were optimized to 30 μL of silver colloid solution and 2 μL of MgSO_4_, which was used to analyze the interaction between GO18 and GTX1/4.

#### 2.2.3. The Interaction between GTX1/4 and GO18

**Figure 7:**
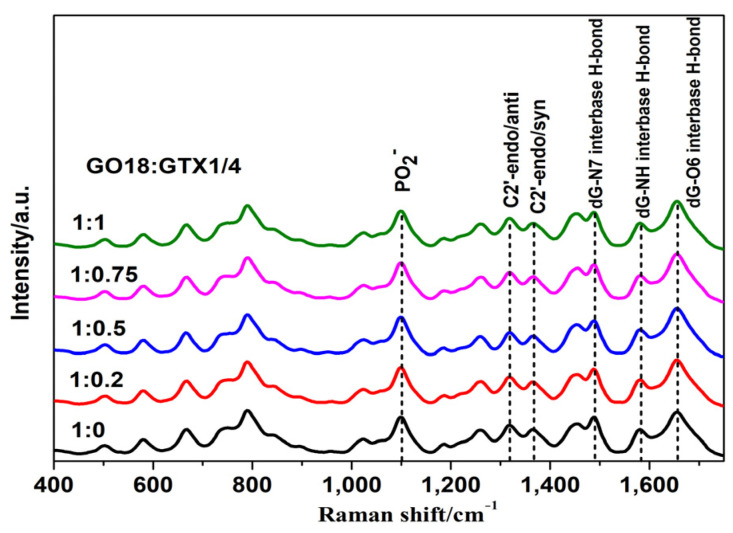
SERS spectra of GO18 at different GTX1/4 ratios.

To study the interaction between GTX1/4 and GO18, SERS spectra of GTX1/4–GO18 solutions at different GTX1/4 ratios were collected (Figure 7). We observed only characteristic signals of GO18 in the SERS spectra but no signals for GTX1/4. This might be because although GTX1/4 and GO18 can bind at a high affinity in the detection system, GO18 was in a free rather than fixed state on the surface of nanoparticles or functionalized nanoparticles, the distance between GTX1/4 and nanoparticles could not be reduced. Thus, the SERS signal of GTX1/4 could not be obtained. During SERS detection of the GTX1/4–GO18 complex, GTX1/4 had no obvious characteristic peak; that is, the GTX1/4–GO18 complex did not constitute signal interference, and the SERS signal observed was only due to GO18 itself before and after combining with GTX1/4. Therefore, it would be helpful to observe the interaction between GTX1/4 and the aptamer, as well as the change in the SERS signal after the interaction.

There was little change in the SERS spectra of aptamers before and after GTX1/4 was added. This might be because under the screening conditions, the aptamer can transform freely and flexibly to undergo an optimal spatial conformation and then combine with GTX1/4. Due to the relatively small molecular volume of GTX1/4, little change was caused due to the conformation of the aptamer, GO18. To analyze this change better, the SERS peak intensity of the PO^2−^ skeleton at 1099 cm^−1^ was used as a standard to normalize the spectra so that the effect of GTX1/4 on the SERS signal of GO18 could be clearly displayed for in-depth analysis.

**Figure 8:**
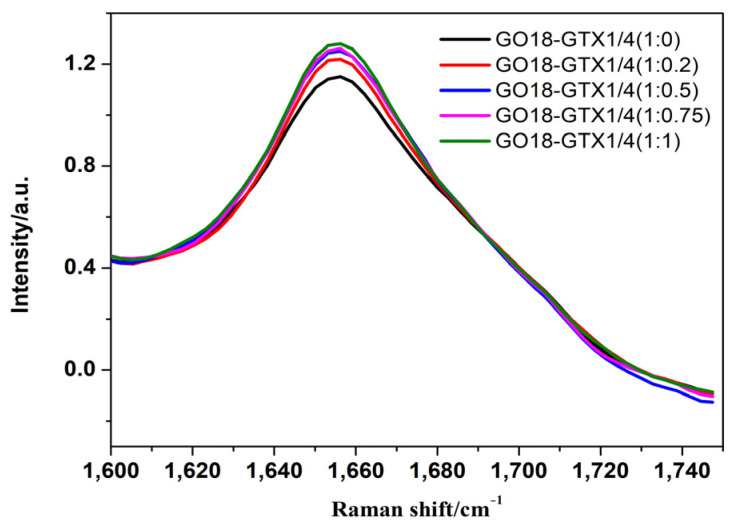
The relative intensity of the peak at 1656 cm^−1^ (I_1656_/I_1099_) under different GTX1/4 ratios.

Some changes were observed in the G-quadruplex correlation bands of DNA. First, Raman-band displacement was observed, and the SERS band near 1487 cm^−1^ indicated that hydrogen bonds were formed between dG (N7) and (N2-H) in the G-quadruplex but not between dG (N7) and water. Concurrently, as shown in Figure S1 (see Supplementary Materials), the peak at 1487 cm^−1^ has a very slight (though not obvious) tendency to shift towards lower wavenumbers. This also indicates that GTX1/4 binds to GO18. The redshift of the SERS spectral band can be attributed to the higher stability of the complex with the increase in GTX1/4 ratio, which is consistent with the findings of previous studies [21]. Second, with the increase in the GTX1/4 ratio in the solution, the intensity of the dG (C6=O6) stretching vibration peak at 1656 cm^−1^ also gradually increased (Figure 8), which further confirmed that the binding sites of GTX1/4 and GO18 were indeed on the G-quadruplex plane. As shown in Figure 1, GTX1/4 combined with the G-quadruplex of GO18, far away from the colloid particle “hot spot,” whereas the negatively charged GO18 molecule was close to the negatively charged silver colloid particles under the action of Mg^2+^; therefore, the SERS characteristic peak of GO18 could still be detected.

**Figure 9:**
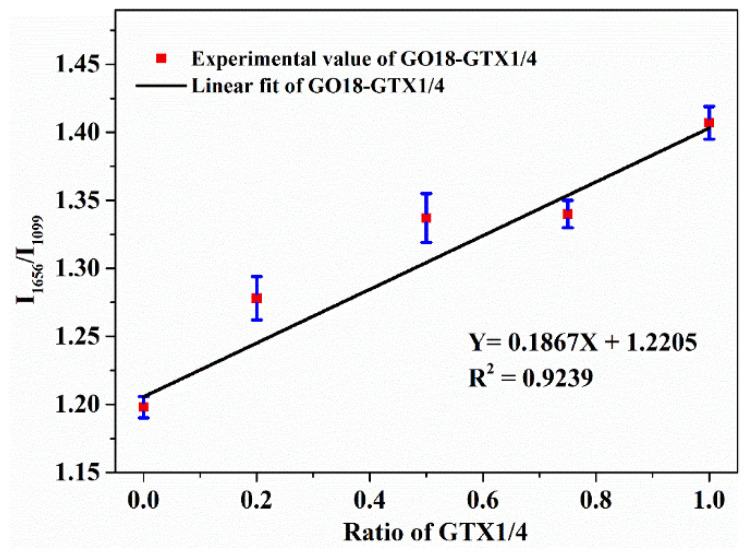
Relationship between the hydrogen bond relative strength of GO18 (I_1656_/I_1099_) and the proportion of GTX1/4.

We then selected the relative intensity of the peak at 1656 cm^−1^ in the SERS spectral band associated with the G-quadruplex in the GTX1/4–GO18 complex (I_1656_/I_1099_) to plot the GTX1/4 ratio in the solution. The results are shown in Figure 9, which demonstrates that they have a certain linear relationship (Y = 0.1867X + 1.2205, R^2^ = 0.9239), indicating that a quantitative relationship could be established between relative peak intensity (I_1656_/I_1099_) and GTX1/4 ratio and suggesting that SERS technology could be used to detect the binding of small ligand molecules with their aptamers.

## 3. Conclusions

This study used GO18 as an example, based on the detection conditions of silver-gel nanoparticles as the reinforcing substrate and MgSO_4_ as the coagulant, explored the influence of the addition of silver colloid solution and MgSO_4_ on SERS detection of ssDNA under four buffer conditions and determined the optimal conditions. The study showed that different buffers had little influence on the detection conditions, and all of them can be used to detect SERS characteristic peaks of ssDNA, which provides a reference method for SERS detection of ssDNA under other buffer conditions and further promotes the research of SERS technology in aptamers. Then, in the Tris-HCl buffer system, the interaction between GTX1/4 and its aptamer, GO18, was studied, which further confirmed that the binding sites of GTX1/4 and GO18 are indeed on the G-quadruplex plane. The quantitative relationship between the relative intensity of the characteristic peaks and the ratio of GTX1/4 was obtained from the measured data (Y = 0.1867X + 1.2205, R^2^ = 0.9239). The results showed that SERS technology could be used to detect the binding of small molecules of the toxin with its aptamer.

## 4. Materials and Methods

### 4.1. Samples and Reagents

GO18 (5′-AACCTTTGGTCGGGCAAGGTAGGTT-3′) was purchased from Sangong Bioengineering Co., Ltd. (Shanghai, China). GTX1/4 was purchased from the National Research Council of Canada. Potassium iodide, magnesium sulfate, sodium acetate, sodium phosphate, silver nitrate, trisodium citrate, and nitric acid were analytically pure and purchased from Sinopril Chemical Reagent Co., Ltd. (Shanghai, China). Other experimental consumables were purchased from the exploration platform. Tris-HCl buffer solution (200 mM, pH 7.5) was purchased from Tianenze Gene Technology Co., Ltd. (Beijing, China). Deionized water was used for all experiments.

### 4.2. Experimental Facilities

A portable Raman spectrometer (K-sens) with an excitation wavelength of 532 nm, a spectral resolution of 6 cm^−1^, and a spectral measurement band of 175–2100 cm^−1^, equipped with a kpRaman-K100 microscope and three magnifying objective lenses (×10, ×20, ×100) and laser beam, with a spot size of 1 μm was purchased from Shanghai Fuxiang Optics Co., Ltd. The high-speed centrifuge (TG16-WS) was purchased from Shanghai Luxiang Centrifuge Co., Ltd. (Shanghai, China). An intelligent magnetic agitator (ZNCL-TS) was purchased from Shanghai Yushen Instrument Co., LTD (Shanghai, China).

### 4.3. Preparation of Silver Colloids

Silver colloids were prepared using a previously published method [37]. Briefly, 194 mL of deionized water was added to a three-necked flask, heated, and stirred, and then 6 mL of 0.6% silver nitrate solution was added, mixed well, heated, and refluxed. When the solution boiled slightly, 4 mL of 1% mass fraction of disodium citrate aqueous solution was added, and the color of the solution changed from colorless and transparent to dark brown and finally turned light yellow and slightly green. After stirring and heating for 60 min, heating was terminated. After cooling to room temperature, the sample was placed in a clean, brown bottle and kept away from light for later use.

Treatment of silver colloids: 10 mL of silver colloid solution was centrifuged (7000× *g*, 10 min). Then, 100 μL of supernatant was removed. Precisely 100 μL of KI solution (1 mM) was added and mixed well, incubated at room temperature for 30 min, and left to stand until use.

### 4.4. Sample Processing

GO18 was dissolved in water, 100 mM sodium acetate (pH 4.5), 100 mM sodium phosphate (pH 7.4), or 100 mM Tris-HCl buffer (pH 7.5) so that the DNA concentration of all solutions was 100 μM and the buffer concentration was 50 mM.

Before SERS detection, all ssDNA samples to be measured were heated in a 90 °C water bath for 10 min, then ice bathed at 0 °C for 5 min, placed at 25 °C for 5 min, and stored in a 4 °C refrigerator so that ssDNA could form a stable G-quadruplex.

### 4.5. SERS Detection Method

SERS conditions were optimized as follows. An amount of 2.5 μL DNA solution and 1, 2, and 3 μL of MgSO_4_ solution at a 0.01 M concentration were successively added to the abovementioned silver-gel solutions of different volumes, and water was added to make final volume of solution 40 μL. SERS was immediately collected after the mixture was evenly mixed. Exactly 10 μL of the solution was placed into a quartz capillary (inner diameter: 0.7 cm, outer diameter: 1.0 cm). The quartz capillary was then placed into the microscope. After the laser was focused on the liquid surface of the capillary, detection was performed using a K-SenS Raman spectrometer (Shanghai Fuxiang Optics Co., Ltd., Shanghai, China).

The spectral detection parameters were as follows: the laser wavelength was 532 nm, the integration time was 20 s, the cumulative number of times was two, and the magnification of the microscope was ×20.

### 4.6. Data Preprocessing

For the collected SERS spectra, spectral band selection (400–1800 cm^−1^) and spectral S-G smoothing (the window size was 9 points, and the curve fitting adopted the second-order polynomial) was performed in sequence. All processing and calculations were completed via a self-written code using Matlab7.0 software, and drawings were established using Origin8.5 software.

## Data Availability

Not applicable.

## Supplementary Materials

The following supporting information can be downloaded at: https://www.mdpi.com/article/10.3390/toxins14010049/s1, Table S1: Intensity of characteristic peak in water. a–c: 4 μL of silver colloids solution; 1, 2, and 3 μL of MgSO_4_, respectively. d–f: 5 μL of silver colloids solution; 1, 2 and 3 μL of MgSO_4_, respectively. g–i: 6 μL of silver colloids solution; 1, 2, and 3 μL of MgSO_4_, respectively. Table S2: Intensity of characteristic peak in sodium acetate solution. a–c: 4 μL of silver colloids solution; 1, 2, and 3 μL of MgSO_4_, respectively. d–f: 5 μL of silver colloids solution; 1, 2, and 3 μL of MgSO_4_, respectively. g–i: 6 μL of silver colloids solution; 1, 2, and 3 μL of MgSO_4_, respectively. Table S3: Intensity of characteristic peak in sodium phosphate solution. a–c: 4 μL of silver colloids solution; 1, 2, and 3 μL of MgSO_4_, respectively. d–f: 5 μL of silver colloids solution; 1, 2, and 3 μL of MgSO_4_, respectively. g–i: 6 μL of silver colloids solution; 1, 2, and 3 μL of MgSO_4_, respectively. Table S4: Intensity of characteristic peak in Tris-HCl. a–c: 5 μL of silver colloids solution; 1, 2, and 3 μL of MgSO_4_, respectively. d–f: 6 μL of silver colloids solution; 1, 2, and 3 μL of MgSO_4_, respectively. g–i: 7 μL of silver colloids solution; 1, 2, and 3 μL of MgSO_4_, respectively. Table S5: Intensity of characteristic peak in Tris-HCl. a–c: 20 μL of silver colloids solution; 1, 2, and 3 μL of MgSO_4_, respectively. d–f: 30 μL of silver colloids solution; 1, 2, and 3 μL of MgSO_4_, respectively. g–i: 40 μL of silver colloids solution; 1, 2, and 3 μL of MgSO_4_, respectively. Figure S1: The relative intensity of the peak at 1487 cm^−1^ under different GTX1/4 ratios.
