# Supplementary material for: Optimization of Surface-Enhanced Raman Spectroscopy Detection Conditions for Interaction between Gonyautoxin and Its Aptamer
Source: Toxins (Basel). 2022 Jan 11;14(1):49. doi: 10.3390/toxins14010049 (PMC8779825; doi:10.3390/toxins14010049)
Supplement: Supplementary file 1 [file toxins-14-00049-s001.zip › toxins-1494460-supplementary.pdf]

toxins

Supplementary Materials: Optimization of surface-enhanced Raman spectroscopy detection conditions for interaction between gonyautoxin and its aptamer

Yan Liu, Lijuan He, Yunli Zhao, Yongbing Cao, Zhiguo Yu and Feng Lu

Table S1. Intensity of characteristic peak in water. a-c: 4 μL of silver colloids solution; 1, 2, and 3 μL of $MgSO_4$, respectively. d-f: 5 μL of silver colloids solution; 1, 2 and 3 μL of $MgSO_4$, respectively. g-i: 6 μL of silver colloids solution; 1, 2, and 3 μL of $MgSO_4$, respectively.

Label	Intensity/a.u.	
	1099 cm^{-1}	1656 cm^{-1}
a	6700.9	5346.7
b	6432.9	4983.2
c	7879.0	6218.8
d	8341.7	6770.5
e*	9375.0	8014.5
f	8216.7	6487.1
g	5636.6	4766.4
h	7413.5	5570.8
i	7325.9	5619.0

Table S2. Intensity of characteristic peak in sodium acetate solution. a-c: 4 μL of silver colloids solution; 1, 2, and 3 μL of $MgSO_4$, respectively. d-f: 5 μL of silver colloids solution; 1, 2, and 3 μL of $MgSO_4$, respectively. g-i: 6 μL of silver colloids solution; 1, 2, and 3 μL of $MgSO_4$, respectively.

Label	Intensity/a.u.	
	1099 cm^{-1}	1656 cm^{-1}
a	4807.7	3892.9
b	5376.3	4938.4
c	3958.9	4566.7
d	4286.9	4807.0
e*	7286.6	8046.3
f	6485.1	4333.3
g	4151.2	5498.6
h	4341.5	6049.6
i	4856.3	4567.2

Table S3. Intensity of characteristic peak in sodium phosphate solution. a-c: 4 μL of silver colloids solution; 1, 2, and 3 μL of $MgSO_4$, respectively. d-f: 5 μL of silver colloids solution; 1, 2, and 3 μL of $MgSO_4$, respectively. g-i: 6 μL of silver colloids solution; 1, 2, and 3 μL of $MgSO_4$, respectively.

Label	Intensity/a.u.	
	**1099 cm^{-1}**	**1656 cm^{-1}**
a	4598.2	3056.3
b	4881.8	3293.0
c	5202.4	4193.8
d	5220.4	4091.6
e	5548.1	3320.6
f	4747.4	3506.4
g	5274.0	3522.6
h*	8512.9	5521.2
i	7010.3	4658.0

Table S4. Intensity of characteristic peak in Tris-HCl. a-c: 5 μL of silver colloids solution; 1, 2, and 3 μL of MgSO4, respectively. d-f: 6 μL of silver colloids solution; 1, 2, and 3 μL of MgSO4, respectively. g-i: 7 μL of silver colloids solution; 1, 2, and 3 μL of MgSO4, respectively.

Label	Intensity/a.u.	
	**1099 cm^{-1}**	**1656 cm^{-1}**
a	4040.1	3064.5
b	5290.8	4104.4
c	6138.9	4983.4
d	7660.2	5159.8
e	7249.2	5284.8
f	5902.5	4373.4
g	4579.8	3639.5
h*	9708.3	6090.3
i	4716.1	3195.2

Table S5. Intensity of characteristic peak in Tris-HCl. a-c: 20 μL of silver colloids solution; 1, 2, and 3 μL of $MgSO_4$, respectively. d-f: 30 μL of silver colloids solution; 1, 2, and 3 μL of $MgSO_4$, respectively. g-i: 40 μL of silver colloids solution; 1, 2, and 3 μL of $MgSO_4$, respectively.

Label	Intensity/a.u.	
	1099 cm^{-1}	1656 cm^{-1}
a	2324.6	3668.0
b	2442.3	3991.0
c	2469.7	4306.8
d	2977.1	4234.9
e*	3429.1	4696.1
f	3414.8	4155.8
g	3539.1	3874.6
h	4084.7	4396.6
i	5232.8	4791.5

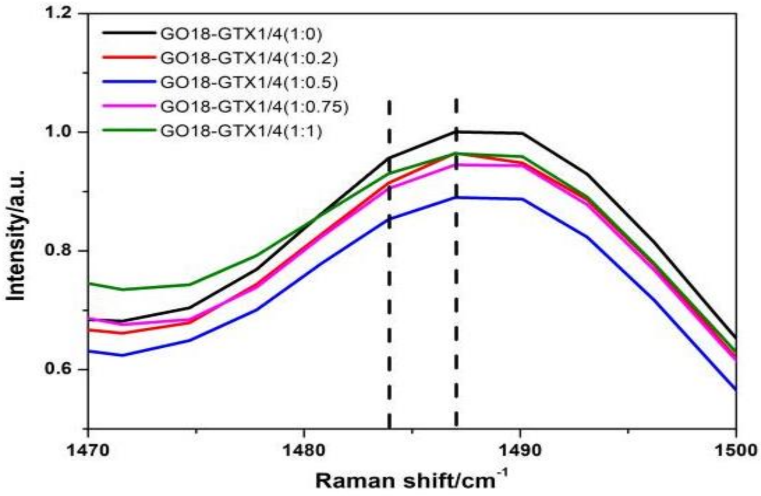

Figure S1. The relative intensity of the peak at 1487 cm^{-1} under different GTX1/4 ratios.
